# Common Genetic Variants in *FOXP2* Are Not Associated with Individual Differences in Language Development

**DOI:** 10.1371/journal.pone.0152576

**Published:** 2016-04-11

**Authors:** Kathryn L. Mueller, Jeffrey C. Murray, Jacob J. Michaelson, Morten H. Christiansen, Sheena Reilly, J. Bruce Tomblin

**Affiliations:** 1 Hearing, Language and Literacy, Murdoch Childrens Institute, Melbourne, Australia; 2 Dept. of Communication Sciences and Disorders, The University of Iowa, Iowa City, United States of America; 3 Dept. of Pediatrics, The University of Iowa, Iowa City, United States of America; 4 Dept. of Psychiatry, The University of Iowa, Iowa City, United States of America; 5 Dept. Psychology, Cornell University, New York, United States of America; 6 Menzies Health Institute, Queensland, Australia; Case Western Reserve University, UNITED STATES

## Abstract

Much of our current knowledge regarding the association of *FOXP2* with speech and language development comes from singleton and small family studies where a small number of rare variants have been identified. However, neither genome-wide nor gene-specific studies have provided evidence that common polymorphisms in the gene contribute to individual differences in language development in the general population. One explanation for this inconsistency is that previous studies have been limited to relatively small samples of individuals with low language abilities, using low density gene coverage. The current study examined the association between common variants in *FOXP2* and a quantitative measure of language ability in a population-based cohort of European decent (*n* = 812). No significant associations were found for a panel of 13 SNPs that covered the coding region of *FOXP2* and extended into the promoter region. Power analyses indicated we should have been able to detect a QTL variance of 0.02 for an associated allele with MAF of 0.2 or greater with 80% power. This suggests that, if a common variant associated with language ability in this gene does exist, it is likely of small effect. Our findings lead us to conclude that while genetic variants in *FOXP2* may be significant for rare forms of language impairment, they do not contribute appreciably to individual variation in the normal range as found in the general population.

## Introduction

Language acquisition requires the interplay of complex biological and behavioural/learning systems, combined with a stimulating and responsive environment where language serves as a tool for social engagement. There is now strong evidence that the neurobiological pathways supporting language learning are genetically influenced (see for instance [1]). Some of the strongest evidence in support of this comes from findings of rare mutations in *FOXP2*.

*FOXP2* was identified via a large multi-generational pedigree—the so-called ‘KE’ family—that appeared to show an unusual autosomal dominant pattern of inheritance for speech and language impairment. Historically, there has been considerable disagreement over how impairments in this family are best characterized. The first published report described “a severe form of developmental verbal apraxia”, since both articulation and expressive language were noted to be affected (p. 352 [2]). The same year Gopnik [3] published work characterising the family’s communication difficulties as “developmental dysphasia” (more commonly known as Specific Language Impairment; p. 715). She described affected family members as “feature-blind”; arguing for a selective grammatical deficit in the use of rule-based morphological paradigms (e.g., the grammatical features that mark tense, number and agreement [3,4]). This was soon contested by evidence showing that affected family members are impaired in aspects of language unrelated to syntax, including phonology and semantics [2,5]. Fletcher [6] proposed a more likely source of the deficits to be in the speech and language production system.

More recent accounts of the KE phenotype have placed greater emphasis on the motor speech aspects of the family’s impairment. The dyspraxic elements first noted by Hurst et al. (1990) affected not just articulation, but also non-linguistic oromotor movements [7–9]. However, they were best exemplified in speech because of the fine-tuned motor movements necessary for oral language. In addition to oromotor weakness, family members presented with mixed dysarthric features [10]. It has been hypothesized that the expressive language impairments (e.g., phonological and syntactic) seen in this family derive from these lower level deficits in oromotor planning and execution [8,11].

Although the literature has primarily focused on expressive language, the KE phenotype is broader and includes deficits in receptive vocabulary [5], grammatical, and syntactic abilities [7]. There is also evidence of cognitive impairment, with more profound deficits in the verbal domain [7]. Because of the involvement of speech, cognitive and motor impairments, not all family members of the KE family would meet the selection criteria for studies of atypical forms of language development (or SLI), as proposed in Gopnik’s original assessment of the family [3, 4]. The discovery of frank neurological dysfunctions among some family members [12] also runs contrary to the current definiton of the disorder [13]. This might lead us to question the relevance of understanding the genetic underpinnings of this severe phenoype for ‘common forms’ of language impairment (e.g., SLI). “However, (it is also possible that) the identification of a specific candidate gene and mutations … can allow the development of targeted investigations in cellular or animal models, which, in turn, can point to mechanisms that might be relevant to more common forms of language-related conditions” (p. 287; [14]).

The initial linkage study of the KE family mapped *FOXP2* to a 5.6-cM region of 7q31 between D7S2459 and D7S643, a region that became known as *SPCH1* (MIM 602081; [15]). Linkage analysis of the family, and mapping of a translocation breakpoint in an unrelated child with a similar phenotype, led to the identification of a gene in this region, *FOXP2* (forkhead box P2; [15,16]). Affected members of the KE family were found to carry a heterozygous point mutation in exon 14 of the gene that was absent in unaffected relatives [16]. This yielded an arginine-to-histidine substitution, R553H (G -> A transition), altering a key residue (Arg^553^). Lai et al. (2001) proposed that the KE phenotype is caused by haploinsufficiency of *FOXP2* during embryogenesis, leading to the abnormal development of neural structures for speech and language [16].

The FOX genes encode a family of transcription factors with a characteristic winged-helix—or forkhead box (“fox”) DNA-binding domain [17]. They regulate a wide variety of cellular and developmental processes, including some in the central nervous system [18]. FOXP2 is highly conserved across species with only three amino acid changes between mice and humans, two of which have occurred in the human lineage since diverging from the chimpanzee [19, 20]. The gene is organized into 19 exons, three of which are alternatively spliced leading to different isoforms [16, 21]. Exons 12–14 encode the DNA-binding domain necessary for transcription factor function [17, 22].

Since the discovery of the KE family mutation, many cases of de novo and familial mutations in *FOXP2* have been reported in the form of point mutations (missense, nonsense and frameshift mutations), small and large scale deletions, sequence alterations; as well as chromosomal alterations, including translocations and genomic copy number variants [23–33]. With such heterogeneity, delineating the precise phenotype(s) associated with the gene is challenging. Individuals with a disruption in *FOXP2* typically present with a severe motor speech disorder, usually verbal dyspraxia. Beyond that, receptive and/or expressive language and/or cognitive abilities and/or more generalised motor skills may also be affected (for a comprehensive review of singleton and family case studies, see [32]). While motor speech impairments seem to be universal in these cases, language impairments are also common and usually considered a core feature of the phenotype [34].

These reports of speech and language impairments in individuals and families with *FOXP2* mutations raise the question as to whether common variants in the gene might be associated with individual differences in the general population. Sequencing of *FOXP2* in children with severe dyspraxia has suggested a low prevalence for etiological variants of approximately 2%; [26]. Studies that have screened moderate-sized samples of children with and without language impairment have found no evidence of a common variant associated with language [35–37].

Despite the lack of positive findings, it would be unwise to reject the possibility that *FOXP2* has a connection to individual differences in language ability on the basis of these mutation searches alone. O’Brien et al. (2003) used sib-pair linkage and family-based association methods to investigate three microsatellites within *FOXP2*; [36]. They found no evidence of linkage or association to SLI as either a binary or quantitative trait. Further sequencing of exon 14 in a subgroup of the sample showed no evidence of functional mutations. Newbury et al. (2002) used a combination of SNPs and microsatellite markers spanning the coding regions of *FOXP2* to investigate quantitative measures of SLI [37]. No mutations were found in the forkhead region of the gene. More recently, however, Rice et al. [38] reported nominally significant associations for four SNPs proximal or within *FOXP2* to a general measure of language ability.

A series of genome-wide linkage and association scans have also failed to detect any signal of association to *FOXP2* for either typical [39, 40] or impaired language abilities [41–52]. Collectively, cohort studies of *FOXP2* suggest that common variants are unlikely involved in more ‘common’ forms of developmental language impairment identified via clinical and population-based samples [36, 37]. However, these studies have been limited in number, by relatively small sample sizes, and by low density gene coverage. They have also focussed only on individuals with impaired speech and language abilities, with unaffected family members comprising the control group.

This study differs from previous research in that, as well as including a large sample of children with language impairment, it also contains a large number of individuals with abilities across the normal spectrum. Thus, it is sensitive to the discovery of a quantitative trait locus. Additionally, this study employed a more extensive panel of tag SNPs to cover the linkage disequilibrium (LD) structure of *FOXP2* than previous studies, including markers in the promoter region of the gene. By comprehensive SNP genotyping of *FOXP2* in a large population-based sample with a continuum of language ability, the current study aimed to address the question of whether *common* genetic variants in *FOXP2* contribute to *individual differences* in language development.

## Results

The primary data for this study came from two earlier studies conducted in Iowa and Illinois. The first group of participants (the Longitudinal cohort) was originally ascertained as part of a cross-sectional study on the prevalence of language disorders in kindergarten [53]. Subsequently, another group (the School-Based cohort) was recruited from a separate study on language abilities in school-aged children. Combined, the total sample comprised 812 children.

All children had been tested for spoken language ability as part of their original study using age-appropriate standardized tests (see Materials and Methods for details). These children represented the full range of spoken language ability, although children with low language abilities were oversampled. As a consequence, the average language ability of the sample in this study was approximately one-third of a standard deviation below the mean (mean Z-score = -.35, SD = 1.10), with a range of -3.35 to 2.59.

Children also provided DNA samples. Thirteen tag SNPs were selected to cover the haplotype block structure of *FOXP2*, and genotyped using Taqman single SNP assays. Details regarding sample recruitment, assessment of language abilities and genotyping methods are detailed further in the Methods Section.

Tests for association to language ability as a quantitative trait (LCOMP; see Methods) consisted of 13 one-way ANOVAs using Proc GLM within SAS, where the genotype at each tag SNP was treated as a class variable. Table 1 summarizes language ability according to genotype at each tag SNP, and the results of the genotype test for association. Overall, we found no statistically significant association between *FOXP2* and language ability (*p* > .05, Table 2). In one case (rs12155328) the nominal *p* level approached significance; however the effect size was quite small, and the *p* level was substantially higher than the .0038 level needed to exceed correction for multiple testing.

**Table 1:** Means and standard deviations (*SD*) of language composite scores for tag SNP genotypes within *FOXP2* and effect sizes (*R*^*2*^) of genotypes on language in the combined Iowa sample.

	G11	G21	G22	R^2^	*F*	*p*
**rs7791396**	C/C	C/G	G/G	0.004	1.60	0.20
**Mean**	-0.38	-0.28	-0.49			
**SD**	1.06	1.13	1.08			
**Count**	295	357	101			
**rs12155328**	C/C	C/T	T/T	0.007	2.38	0.09
**Mean**	-0.36	-0.23	-0.46			
**SD**	1.10	1.12	1.08			
**Count**	242	326	161			
**rs10447760**	C/C	C/T	T/T	0.0005	0.17	0.84
**Mean**	-0.38	-0.33	-0.39			
**SD**	1.07	1.11	1.07			
**Count**	446	269	51			
**rs10953754**	A/A	A/G	G/G	0.00007	0.03	0.97
**Mean**	-0.36	-0.35	-0.38			
**SD**	1.10	1.07	1.15			
**Count**	280	373	111			
**rs2244419**	C/C	C/T	T/T	0.0004	0.15	0.86
**Mean**	-0.34	-0.39	-0.21			
**SD**	1.11	1.03	0.77			
**Count**	676	114	4			
**rs1668335**	AA	A/G	GG	0.001	0.46	0.63
**Mean**	-0.26	-0.32	-0.37			
**SD**	1.11	1.12	1.07			
**Count**	78	352	359			
**rs2396720**	A/A	A/C	C/C	0.0009	0.71	0.40
**Mean**		-0.15	-0.40			
**SD**		1.03	1.09			
**Count**	0	23	768			
**rs1916988**	C/C	C/T	T/T	0.0003	0.13	0.87
**Mean**	-0.30	-0.35	-0.36			
**SD**	1.04	1.10	1.10			
**Count**	76	354	348			
**rs11505922**	C/C	C/T	T/T	0.0008	0.32	0.73
**Mean**	-0.31	-0.35	-0.40			
**SD**	1.10	1.10	1.08			
**Count**	177	399	215			
**rs7785701**	C/C	C/G	G/G	0.0005	0.22	0.80
**Mean**	-0.32	-0.32	-0.38			
**SD**	1.06	1.12	1.09			
**Count**	165	371	238			
**rs2106900**	A/A	A/G	G/G	0.001	0.45	0.64
**Mean**	-0.37	-0.30	-0.38			
**SD**	1.01	1.10	1.06			
**Count**	170	360	241			
**rs7799652**	G/G	G/T	T/T	0.003	1.18	0.31
**Mean**	-0.26	-0.39	-0.28			
**SD**	1.10	1.10	1.06			
**Count**	179	370	206			
**rs1005958**	C/C	C/T	T/T	0.002	0.92	0.40
**Mean**	-0.29	-0.32	-0.43			
**SD**	1.11	1.08	1.08			
**Count**	161	380	218			

**Table 2:** Means and standard deviations of language composite scores by genotype at rs1916988.

Sample	rs1916988 genotypes			*R*^*2*^	*F*	*p*
	CC	TC	TT			
**Longitudinal**						
**Mean**	-0.63	-0.60	-0.41	0.0078	1.89	0.16
**SD**	(1.07)	(1.07)	(1.02)			
**Count**	42	211	219			
**School**						
**Mean**	0.16	0.03	-0.28	0.03	4.24	0.015
**SD**	(0.83)	(1.04)	(1.02)			
**Count**	33	143	129			
**ELVS**						
**Mean**	-0.23	-0.11	0.02	0.0082	1.23	0.29
**SD**	(1.10)	(1.02)	(0.80)			
**Count**	31	144	133			

The data above were based on the Longitudinal and School-Based samples combined. Although the phenotype measures overlapped in these two cohorts and prior research based on the Longitudinal sample has demonstrated that all measures for the two cohorts are highly correlated [54], the two groups were ascertained differently. The Longitudinal cohort over-sampled children with low language ability, whereas the School-Based cohort was truly a population sample. These differences resulted in the Longitudinal sample having a lower average language ability level (*M* = -0.51, *SD* = 1.11) than the School-Based one (*M* = -.09, *SD* = .99). Thus, it remained possible that combining groups might obscure statistically-significant associations.

Therefore, the data were analysed for an interaction of genotype effects at each tag SNP with sample membership. One SNP yielded a significant genotype by phenotype interaction (rs1916988: *F*(2,772) = 5.76, *p* = .003) after adjustment for multiple testing (see Table 2). A test for simple effects of genotype by cohort showed a significant genotype effect in the School-Based sample, *F* = (2, 302) = 4.24, *p* = .015. There was no significant effect of this SNP in the Longitudinal sample. A comparison of genotype means in the School-Based sample (Table 2) showed that the TT genotype group had significantly lower language abilities than the TC group (*p* < .05), suggestive of a dominance effect for the C allele. By comparison, in the Longitudinal sample, the TT group averaged higher scores than groups carrying the C allele, although these were non-significant. Thus, the direction of the effect in the two samples was in the opposite direction.

These results leave the status of association between rs1916988 in *FOXP2* and individual differences in language ability unclear. The School-Based sample that yielded a significant association had a distribution of language abilities that was very similar to the normative samples used in the design of the designated language tests, whereas the Longitudinal sample comprised an excess of children with poor language abilities. It is therefore possible that the strength association is dependent upon the overall level of language ability in the sample tested.

In order to resolve this ambiguity, we obtained data from a third sample of participants in a longitudinal birth cohort study (Early Language in Victoria Study: ELVS; [55]). ELVS was a population sample assessed for language ability with a subset of the same measures used in the Iowa Longitudinal and School-Based samples.

Mean z-scores for the ELVS’ sample by genotype are shown in Table 2. A test for genotype effects at rs1916988 showed no differences between mean language scores across the three genotype groups, *F*(2, 305) = 1.23, *p* = .29. Thus, these data are consistent with the results of the Longitudinal sample. The effect sizes for the ELVS and Longitudinal samples were similar and the direction of the effects, albeit non-significant, was the same. When the Longitudinal and ELVS samples were combined via meta-analysis, the weighted *R*^*2*^ effect size was .086. However, the lower bound of the 95% CI was -0.009 and the upper bound was 0.18. Thus, even in the combined samples, the effect was small and non-significant.

In order to assess whether a combination of SNPs were predictive of the language phenotype in a multivariate setting, we also fit a predictive model using Random Forests (RF; [56]). In essence, this analysis fits decision trees by splitting the data (i.e. the individuals) recursively based on genotypes at the different SNPs. In doing this, it aims to group individuals with similar language scores together. Data are divided into a training set and a test set, resulting in a less biased estimate of the predictive power of the RF. Random Forests have been repeatedly used in such genetic association settings, especially where genetic interactions (epistasis) are of interest [57–59]. The advantage to RF is that it looks at all SNPs simultaneously, rather than in isolation, and if there are interactions between SNPs, or other kinds of combinations that are predictive, these will be detected.

When using LCOMP as the response variable (quantitative outcome), the correlation between the prediction on an independent test set (i.e., subjects held out of the training set) and the actual LCOMP values was *r* = -.0178, 95% CI (-0.09, 0.05), *p* = 0.61; power = 0.8 for *r* = 0.115 at α = 0.05). RF also has a built-in measure of variable importance, which can be used as an indicator of how much predictive power a SNP carries alone or in combination (e.g. epistasis) with other SNPs. No SNP had an importance score significantly greater than those obtained through permutation of the data. These results were robust even in the face of RF parameter tuning (RF typically needs little or no parameter tuning for optimal performance). Taken together, these results suggest that even in a multivariate machine learning paradigm, SNPs in *FOXP2* have little or no explanatory power for language phenotypes in our sample.

## Discussion

This is the first study to investigate the association of common variants in *FOXP2* to individual differences in language ability in a large sample with a range of language abilities. Much of our current knowledge regarding the neural correlates of *FOXP2* comes from intensive study of a single multiplex family (the ‘KE’ family) that display an unusual speech and language phenotype due to a missense mutation in the FOX domain. Etiological point mutations and gross chromosomal rearrangements (e.g., deletions and translocations) have also been reported in singletons and small family studies [23–33].

A few studies have considered whether common variants in *FOXP2* are associated with language impairment (e.g., [37, 38]). However, these have been limited with regards sample size, comprising a relatively small number of affected individuals and their family members. O’Brien et al. (2003) have previously tested for association of common variation within *FOXP2* and a sample with a range of language abilities (i.e., the Longitudinal cohort 1 in the current study); however coverage of the gene was limited [36].

In this study, we considered the full range of language abilities existent in the general population of unrelated individuals, and selected tag SNPs to cover the majority of LD structure found in *FOXP2*. We genotyped 13 common polymorphisms in 812 individuals, testing for association to a quantitative measure of language ability, with null results. One SNP provided evidence of an association in a subgroup of the participants in this study; however, these findings did not replicate. In conjunction with previous research indicating the rare and specific nature of *FOXP2* mutations in the etiology of speech and language disorders, these findings lead us to conclude that common variants are unlikely to exert a large effect in typical language development. Using the combined longitudinal and school samples this study was powered to detect a QTL variance of 0.02 for an associated allele with MAF of 0.2 or greater with 80% power [60]. This means we had sufficient power to detect genetic effects responsible for at least 2% of the variance in our composite measure of language ability. Furthermore, this study had the additional advantage of an independent sample to test for replications of any positive findings. Our largest effect size (R^2^) for the combined sample was 0.001; thus it is possible that small genetic effects from *FOXP2* contributing to individual differences in language ability may exist. One possibility is that the SNPs within *FOXP2* each contribute some unique effects and that a combination of these effects could be large enough to be detectible; however our use of Random Forest regression analysis did not yield any significant evidence of such effects. If so, these effects are likely very small and would therefore be a part of a large ensemble of polygenetic background for individual differences in language.

In spite of this being one of the largest studies of its kind, we may still have been underpowered to detect common risk variants in *FOXP2* of small effect size (i.e., those which affect less than 2% of variance in the composite language measure). This issue could be addressed by screening SNPs in a larger sample size, which would boost power. Also, with the advent of cost-effective whole exome and whole genome sequencing, it should soon be possible to determine the population effect of rare variants (i.e., infrequent alleles of large effect) in *FOXP2* for phenotypes involving speech and language.

Nevertheless, *FOXP2* has been critical in providing ‘a molecular window’ into the genetic bases of speech and language impairments [34] in that identification of the gene has opened up avenues of investigation into signaling pathways [61–64]. In part, this is because *FOXP2* serves as a regulatory gene—whose primary role is to modify the timing and expression of downstream genetic targets [17]. As such, it likely represents one of many elements in gene networks involved in speech and language development

This role for *FOXP2* as a regulator in a network of genes important for language is demonstrated by evidence showing that up-regulation of *FOXP2* coincides with the down regulation of expression in another gene in the 7q region, *CNTNAP2* [64, 65]. *CNTNAP2* encodes CASPR2, a neurexin found at the nodes of Ranvier in myelinated nerve fibers. It is expressed in the human cerebral cortex, specifically the frontal and temporal lobes and the striatum [66]; regions that are important for language and cognition [67]. Common polymorphisms in *CNTNAP2* have been associated with language delay in autism [66] and the general population [68]; and more specifically to phonological memory [65] and reading abilities in language impairment [69, 70].

The null findings from this study may have implications for the study of the evolutionary properties of *FOXP2*. These data suggest that the mutations in *FOXP2* with negative functional consequences may be under considerable selection pressure. Whether this selection is based on poor language or other concomitant functions is not clear. A study by Ayub et al. (2013) investigated whether recent positive selection on *FOXP2* is also associated with positive selection on any known target genes [18]. They examined four different populations and found strong evidence for selection in Europeans, but not in the Han Chinese, Japanese or Yoruba populations. This may suggest selection of *FOXP2* targets has occurred fairly recently, after the divergence of the populations, from local adaptation.

This study failed to reject the null hypothesis that common polymorphisms in *FOXP2* are associated with population differences in language ability, building on previous research by examining coding regions in the 5’ promoter region of the gene that could affect transcription factor binding. However, sequence analysis of *FOXP2* indicates a promoter region flanking exon s1 upstream of the gene [21], and it is entirely possible that our approach to genotyping failed to detect a putative signal from this region. Therefore, we cannot exclude the possibility that regulatory processes governing the expression of *FOXP2* are important for individual differences in language development. This is important because *FOXP2* expression levels in turn affect the expression of putative target genes, including those involved in neurite outgrowth and striatal plasticity [63, 71]. Gene knock-in of the humanized version of *FOXP2* to mice has been found to specifically affect cortico-basal ganglia circuits (including the striatum; [72]), and facilitate both declarative and procedural learning [73]—two learning processes thought to be crucial for language acquisition.

Ultimately, the aim of future research into *FOXP2* will be to characterize the regulatory networks or pathways of which the gene is a part, the implications of these for cellular and neuronal processes (for example, synaptic plasticity), and the role of these in shaping the mechanisms for language learning.

## Materials and Methods

The study was approved by the Institutional Review Board at the University of Iowa, which subscribes to the basic ethical principles underlying the conduct of research involving human subjects, as laid out in "The Belmont Report". Parents provided written consent for their children’s participation in the project and for use of their DNA.

Participants were a sub-set of two larger studies on childhood language development and disorders.

### Longitudinal Sample

#### Participants

The Longitudinal cohort (*n* = 500) was initially ascertained as part of a cross-sectional study on the prevalence of language disorders in kindergarten (7,218 participants screened; [53]); and were subsequently enrolled in a longitudinal study of outcomes in children with and without language impairment (see [74]). All children in this sample were mono-English speakers, had normal hearing and no reported neurodevelopmental disabilities. Because the longitudinal study was concerned with language impairment, it was intentionally designed to oversample for children with poor language abilities. To correct for this in the current study, we employed a weighting system. Children were assigned a weight value that represented the reciprocal of the probability that the child would be sampled from the original population. Children with high probabilities were given low weighting values, and children with low probabilities were given high values. This has the effect of reducing the contribution of children with language impairment to the study norm, and means the study sample approximates the original cross-section sample from which it was drawn (see [54] for details of this weighting method).

#### Language Phenotype

The phenotype employed in the Longitudinal sample (see Table 2) is based on a scheme proposed by Tomblin et al. (1996; [75]). It comprised five subtests from a standardized language measure, the *Test of Language Development-2*:*P* (TOLD-2:P; [76]), and a narrative production and comprehension screen [77]. The subtests were selected to represent norm-based performance across three domains (vocabulary, grammar, and narration) and two modalities (comprehension and expression) of language. Raw scores were converted into standard scores based upon local norms [75] and combined to form an overall language composite. Factor analysis of these five measures of language showed that a single factor accounted for co-variance among the measures [78]. Thus, a composite score can be used as an appropriate representation of the language phenotype. This has the advantage of limiting the number of inferential tests, and enhancing reliability.

Participants also completed the Block Design and Picture Completion test of the *Wechsler Preschool and Primary Scale of Intelligence* (WPPSI; [79]). These tests of nonverbal (or performance) IQ were chosen to prevent confound with language abilities, as assessed by verbal and total IQ scores. Any proband with a nonverbal IQ <70 was excluded from the study on the basis of intellectual disability.

### School Sample

#### Participants

In addition to the prevalence/longitudinal sample described above, a separate group of participants (*n = 318*) were recruited in 2007/2008 from a study on language abilities in school-aged children.

#### Language Phenotype

Children in participating schools in grades one to four were screened using the verbal subscales of the *Iowa Tests of Basic Skills* [80], which have been found by our laboratory to be good predictors of receptive and expressive language abilities. Children with scores suggestive of poor language abilities, along with a random sample likely to have normal language, were then administered a more comprehensive test battery for their age (see Table 3). Again, all children were tested for normal hearing and according to parent report had no neurodevelopmental disability. The assessment of language ability in the School-Based sample paralleled that of the Longitudinal sample, although specific measures were changed to reflect the different age levels of participants [81, 82]. A composite language score (LCOMP) was derived in the same way as for the Longitudinal sample, although scores were not weighted. Similarly, participants also completed a nonverbal IQ test [83]. Again, any proband with a nonverbal IQ <70 was excluded from the study on the basis of intellectual disability.

**Table 3:** Language measures in each sample.

Language Domain	Sample		
	Longitudinal (5–7 years)	School (7–9 years)	ELVS (7 years)
**Vocabulary**	**TOLD-2: P**	**PPVT-R**	**PPVT-IV**
	Picture Vocabulary		
	Oral Vocabulary		
**Sentence**	**TOLD-2: P**	**CELF-III**	**CELF-III**
	Grammatic Understanding	Sentence Structure	Sentence Structure
	Grammatic Completion	Concepts & Directions	Concepts & Directions
	Sentence Imitation	Recalling Sentences	Recalling Sentences
		Word Structure	Word Structure
**Narrative**	**Culatta, Page & Ellis (1983)**	**CELF-III**	
	Story Retell	Recalling Paragraphs	
	Story Comprehension	Story Generation	
**Non-verbal IQ**	**WPPSI**	**WISC-III**	
	Block Design	Block Design	
	Picture Completion	Picture Completion	

TOLD-2:P = Test of Oral Language Development-2:Primary; PPPVT-R = Peabody Picture Vocabulary Test-Revised; PPVT-IV = Peabody Picture Vocabulary Test, 4^th^ Edn; CELF-III = Clinical Evaluation of Language Fundamentals-III; Story Retell and Story Comprehension = Culatta, Page & Ellis, 1983; WPPSI = Weschler Preschool Primary Scales of Intelligence; WISC-III = Wechsler Intelligence Scales for Children-III.

### ELVS Sample

#### Participants and Phenotypes

Participants in the ELVS sample were part of a birth cohort (Early Language in Victoria Study, N = 1,910) recruited in and around the metropolitan area of Melbourne, Australia. Data for the current study was obtained when children were around seven years of age. Consent for participation was obtained from the parents of all children and the children also assented to participate. As a part of the 7-year wave of assessment, participants provided DNA from saliva samples. As with the Iowa sample, all children were of European ancestry and had no developmental disabilities or hearing loss. Measures of language ability were age appropriate measures of listening and speaking similar to those used in the Iowa Longitudinal and School-Based samples (Table 3; [82, 84]). Again, a composite score was derived to represent the children’s overall language ability. DNA and language phenotypes were available for 308 participants in the current study.

Participants from all cohorts were monolingual speakers of English with normal hearing and without any comorbid neurodevelopmental disorders (e.g., autism), based on parental report.

### DNA Processing and Genotyping

DNA for the Iowa cohorts was obtained for 818 probands from blood, buccal swabs and saliva, and processed using standard protocols. DNA for the ELVS’ group (*n* = 308) comprised saliva samples only. Briefly, DNA was extracted from buccal swabs using procedures described in Richards et al. [85], and from whole blood using a modified version of Qiagen’s DNA Blood Maxi Kit (Qiagen, Inc., Valencia, CA). Samples derived from saliva were processed using Oragene’s standard 0.5mL and 4mL protocols (DNA Genotek, Ontario, Canada).

**Fig 1:**
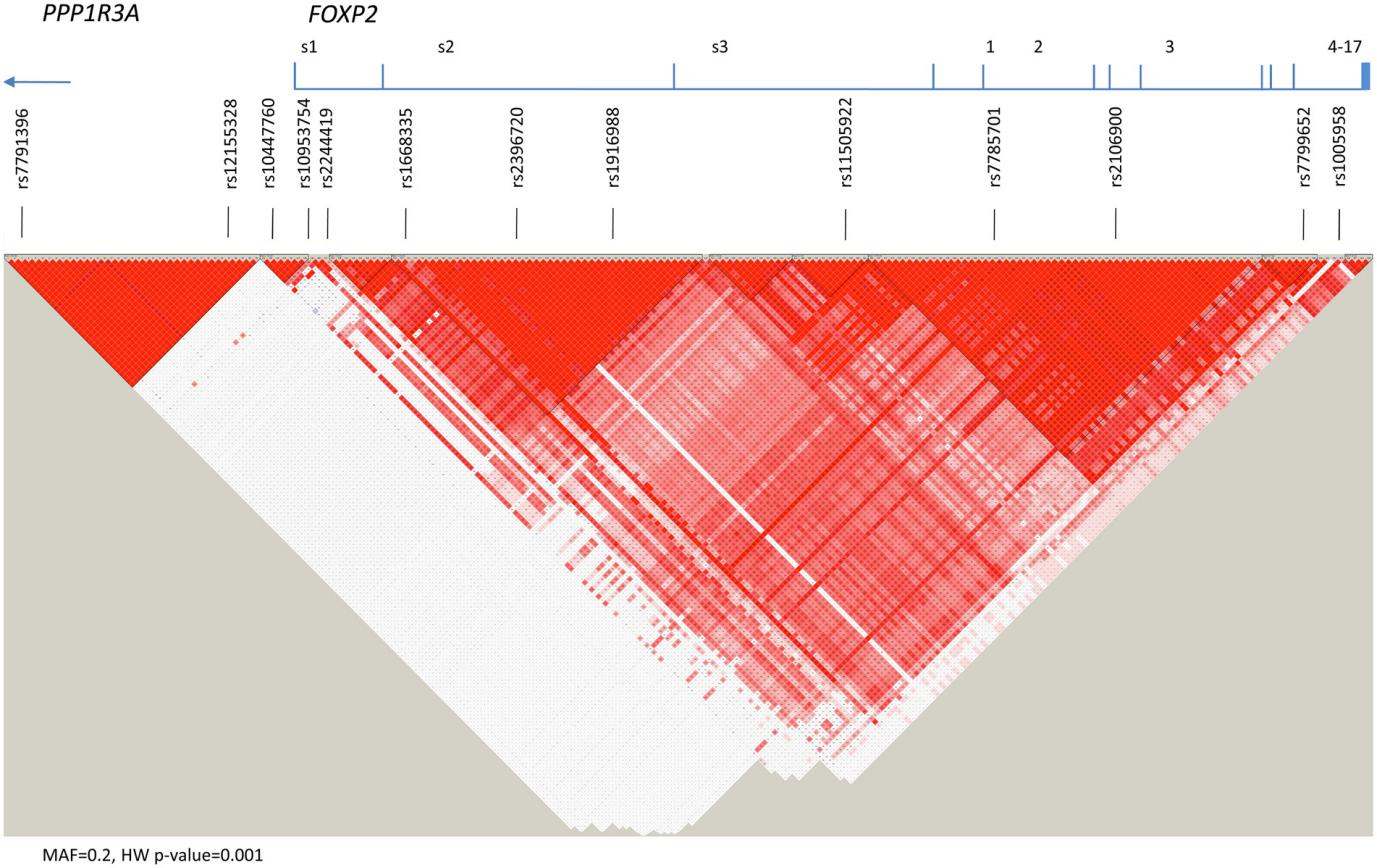
Linkage disequilibrium structure of FOXP2 with candidate SNPs.

Note: LD structure from http://www.hapmap.org. Base-pair location is based on the National Center for Biotechnology Institute db SNP Build 131 of the human genome (NCBI dbSNP 131).

Single nucleotide polymorphisms (SNPs) were selected to cover the haplotype block structure of *FOXP2*, including exonic regions of high linkage disequilibrium in the 5’ promoter and 3’ ends of the gene (Fig 1; [86–89]). We included variants in the promoter region of the gene because these can result in different isoforms, which affect the amount and timing of protein production during gene expression. Sequence analyses have provided evidence for at least one promoter region flanking exon s1 of the gene, located more than 300kb 5’ to exon s1 as first described by Bruce and Margolis [23].

SNPs were selected on the basis of minor allele frequency (MAF) greater than 0.2 in the CEPH population (Utah Residents with Northern and Western European Ancestry; Table 4 The International HapMap Project). Selection of the correct reference population is an important design consideration because alleles and haplotypes vary by ethnicity [90]. The rationale for using a minimum MAF of 0.2 meant we should have been able to detect a QTL variance of 0.02 for an associated allele with MAF of 0.2 or greater with 80% power in our study population. Exceptions to this are rs2244419 and rs2396720, which have lower MAFs, but were chosen only after assays in that region of linkage disequilibrium (LD) failed quality control (QC) standards. A senior geneticist, with extensive experience in this area, approved all SNP selections.

**Table 4:** SNP location, minor allele frequency and call rates for markers in *FOXP2*.

SNP	Location	MAF	Alleles*	SNP call rate		
				Longitudinal	School	Combined
**rs7791396**	113,414,165	.3744	G/C	.92	.94	.93
**rs12155328**	113,489,236	.4471	T/C	.87	.94	.9
**rs10447760**	113,510,501	.243	T/C	.92	.97	.94
**rs10953754**	113,550,510	.3924	G/A	.93	.96	.94
**rs2244419**	113,579,759	.08037	T/C	.98	.98	.98
**rs1668335**	113,647,888	.3245	A/G	.96	.99	.97
**rs2396720**	113,688,077	.0148	A/C	.96	.99	.97
**rs1916988**	113,716,698	.3279	C/T	.95	.98	.96
**rs11505922**	113,797,887	.4796	C/T	.97	.97	.97
**rs7785701**	113,856,772	.4465	C/G	.94	.98	.95
**rs2106900**	113,909,742	.456	A/G	.94	.97	.95
**rs7799652**	114,077,719	.4832	G/T	.91	.95	.93
**rs1005958**	114,090,091	.4635	C/T	.92	.96	.93

* The A1 allele is the minor allele. Base-pair location is based on the National Center for Biotechnology Institute db SNP Build 131 of the human genome (NCBI dbSNP 131).

Because allele frequencies can vary by population, we aimed to minimize potential confounds arising from genetic ancestry by genotyping only samples from Caucasian individuals. Ethnicity was determined via parental report. Allelic variation was determined via the Sequence Detection Systems 2.2 software (SDS, Applied Biosystems). Genotype data was uploaded into a Progeny database (Progeny Software, LLC, South Bend, IN) and integrated with phenotypic information via Microsoft Access.

### Quality Control and Statistical Analyses

DNA samples can vary in both quality and concentration, affecting both genotype call rate and accuracy. DNA that is poor quality results in low call rates and inaccuracies in genotyping. Initial quality control steps included both biological and technological quality checks. Prior to genotyping, biological samples were quantified using The Thermo Scientific NanoDrop^™^ 1000 Spectrophotometer. Samples that were suspected to be contaminated, and those of low grade quality, were excluded from the study. Taqman Pre-designed Genotyping Assays (Applied Biosystems, Foster City, CA, USA) were tested prior to use with CEPH control plates.

Genotyping was performed using Taqman Pre-designed Genotyping Assays under standard reaction conditions (Applied Biosystems, Foster City, CA, USA). Genotypes for each SNP and each individual were called using the algorithm in SDS, which is based on the relative signal intensity of each allele for each SNP. Plots were then inspected manually by a lab technician with special expertise in this area. Low quality genotype calls were excluded from data analysis. There was no reassignment of SNP genotypes.

Further steps in quality control were performed using PLINK (v1.07; [91]). The first of these involved calculating SNP call rates. The call rate per SNP is the percentage of individuals whose genotypes are called for a given SNP. It is an important part of quality control because low call rates can lead to inaccuracies in genotype calls.

Table 4 details chromosomal location, alleles, minor allele frequencies, and call rates for the SNPs genotyped in this study. In general, call rates were higher in the School-Based Sample than the Longitudinal Sample. With the exception of rs12155328 in the Longitudinal cohort, all SNP call rates were ≥ 90% (see Table 4 for call rates of individual SNPs).

Another indicator of data quality is heterozygosity. An excessive or reduced proportion of heterozygous genotypes may be indicative of sample contamination or of inbreeding. The heterozygosity of samples was in this study .40, compared to .41 in the reference dataset (CEU population, NCBI Genome Build 131), which is not statistically significant.

Hardy Weinberg Equilibrium (HWE) may also be used as a measure of the fidelity of genotyping. Genotyping error is indicated by SNPs that show significant deviation from HWE, based on a pre-specified level of significance (p ≤ .001). However, such deviation may also be a signal of genetic association. For this reason, HWE was tested only in the control samples. There was no significant deviation from HWE in any of the cohorts.

Tests for association to language ability as a quantitative trait comprised 13 one-way ANOVAs using Proc GLM within SAS, where the genotype at each tag SNP was treated as a categorical variable. In addition to performing univariate statistical association tests, we also examined whether the genotyped SNPs were predictive of language phenotypes in a multivariate setting. It is conceivable that SNPs interact or otherwise show combined predictive power that might not be uncovered by traditional tests of association. To examine this possibility, we used Random Forests [56], a machine learning approach that fits an ensemble of decision trees to the data. In this setting, the SNP genotypes were the predictor variables, and the quantitative LCOMP trait was used as the response or outcome variable. These analyses were performed using R (www.r-project.org) version 3.2.3 and the R randomForest package version 4.6–12.
